# Editorial: Vitamin D: from pathophysiology to clinical impact, volume II

**DOI:** 10.3389/fnut.2024.1506137

**Published:** 2024-10-29

**Authors:** Cristina Vassalle

**Affiliations:** Fondazione G Monasterio, Fondazione CNR-Regione Toscana G Monasterio, Pisa, Italy

**Keywords:** 25(OH)D, extraskeletal districts, reference levels, threshold, vitamin D

Besides the well-known positive effects on skeletal homeostasis and bone metabolism, growing evidence highlights the importance of vitamin D also in other many extra-skeletal districts; the articles published in this Research Topic confirm this observation in both adult and pediatric populations, spanning different conditions from inflammation and infectious diseases, obesity, and diabetes, to neurological disorders, gastrointestinal conditions, neurological disorders, cardiovascular health, and malignancies (see Table 1). This fact contributes to the increasing attention toward the measurement of serum 25(OH)D (main circulating form and recognized biomarker of vitamin D status) in laboratory medicine as well as the requirements of accuracy and speed of testing. However, despite the growth and refinement of analytical methods, the measurement of vitamin D still represents a challenge; immunoanalytical techniques still retain great variability in inter-laboratory comparisons, whereas mass spectrometry presents many different difficulties (e.g., costs, time, and complexity, matrix effects, derivatization step) (1). Interestingly, other vitamin D metabolites may have biological roles, and are expected to be assessed in the serum in the future (2).

**Table 1:** Contributions of the Research Topic.

**Title**	**Authors**	**Setting**	**Patients**	**Results**
No causal relationship between serum vitamin D levels and alcoholic liver disease: a two-sample bidirectional Mendelian randomization study	Wu H, Wu L, Zhang Q, Li C, Li HY, Zhang BF.	Alcoholic liver disease (ALD)	Adults	No reciprocal causal link between serum VD and ALD susceptibility
Vitamin D in tuberous sclerosis complex-associated tumors	Tatsuro Nobutoki	Tuberous sclerosis complex-associated tumors	Children	Review discussing the possible role of 1,25VD in pediatric tuberous sclerosis complex-associated tumors, and the significance of vitamin D signaling as adjuvant or alternative drug target
Vitamin D status, vitamin D receptor, CYP2R1, and CYP24A1 profiles in children	Iriani A, Rachman A, Fatina MK, Gemilang RK, Trisnandi A, Muskananfola FV, Nugraha MFI.	Vitamin D status	Children	VD level is reduced by aging in children; VDR level is also found different based on VD status
Explorative case control study on the associations of serum vitamin D3, folic acid and vitamin B12 levels on Kawasaki disease and coronary artery lesions	Chen Y, Liu X, Li B, Li J, Meng L, Ye C, Han L, Li H, Deng LL, Su Z, Zhang X.	Kawasaki disease and coronary artery lesions	Children	Folic acid and VD3 were significantly reduced in children with KD, especially in those with coronary artery lesions
Changes in vitamin D status among adults from the COVID-19 pandemic to post-pandemic normality	Chen Y, Kong G.	COVID-19	Adults	COVID-19 pandemic significantly impacted VD status, leading to an increased prevalence of deficiency, especially among males
Nonlinear correlation and mediation effects between serum 25-hydroxyvitamin D levels and all-cause mortality in COPD patients	Jiang Q, Jiang Y, Ma Z, Huang J, Li Y.	COPD	Adults	High prevalence of low VD in COPD patients; non-linear association between serum 25(OH)D concentration and all-cause mortality (better survival above 63.40 nmol/L), with VD mediating role between dietary inflammation and mortality (mediation analysis)
The effects of vitamin D supplementation on serum lipid profiles in people with type 2 diabetes: a systematic review and meta-analysis of randomized controlled trials	Lu Q, Liang Q, Xi Y.	Type 2 diabetes	Adults	The meta-analysis evidenced the benefit of VD supplementation in improving serum HDL and TG but not in LDL and TC levels
Associations of vitamin D status with all-cause and cause-specific mortality in long-term prescription opioid users	Dai S, Wu J, Wang P, Hu Z.	Opioid users	Adults	Non-linear association between serum 25(OH)D concentration and all-cause mortality
Implications of vitamin D levels or status for mortality in rheumatoid arthritis: analysis of 2001-2018 data from the National Health and Nutrition Examination Survey	Feng Y, Zhu P, Dandan Y, Wang X, Chen C, Zhang Z, Tian Y, Wang J, Liu S, Li J, Meng D, Wang K.	Rheumatoid arthritis (RA)	Adults	A significant negative correlation between 25(OH)D levels and overall mortality in individuals with RA, especially for mortality related to heart disease and cancer
Serum 25-hydroxyvitamin D concentrations and their impact on all-cause mortality in Parkinson's disease: insights from National Health and Nutrition Examination Survey 1999–2020 data	Yong Y, Dong H, Zhou Z, Zhu Y, Gu M, Li W.	Parkinson's disease	Adults	Non-linear association between serum 25(OH)D concentration and all-cause mortality (optimal survival rates at 75–100 nmol/L)
The complex relationship between vitamin D and kidney stones: balance, risks, and prevention strategies	Zhang F, Li W.	Kidney stones	Adults	The review discusses actual evidence on the relationship between vitamin D status and kidney stone risk, and the role of vitamin D supplementation for preventing and treating kidney stones.
Factors associated with vitamin D deficiency in health care workers exposed to SARS-CoV-2: a cross-sectional study	Villasis-Keever MA, Zurita-Cruz JN, Garduño-Espinosa J, López-Alarcón M, Barradas Vázquez AS, Miranda-Novales MG, Parra-Ortega I, López-Martinez B, García H, Klünder-Klünder M.	COVID-19	Adults	High prevalence of VD deficiency in Mexican health care workers exposed to SARS-CoV-2, related to both personal health factors and occupational low VD values is high among health care workers, and TD2 was a significant risk factor associated with these values. conditions (e.g. T2D)
The relationship between physical activity levels and serum vitamin D levels varies among children and adolescents in different age groups	Ouyang S, Li Q, Liu Z, Yin Y.	Physical activity (PA)	Children and adolescents	PA and VD varies according to sex and age, with sun exposure level and BMI affecting PA/VD relationship
Comparative Analysis of COVID-19 Responses in Japan and Africa: Diet, Phytochemicals, Vitamin D, and Gut Microbiota in Reducing Mortality -A Systematic Review and Meta-Analysis	Santa K, Tamaki R, Watanabe K, Nagaoka I.	COVID-19	Adults	Blood vitamin D levels are associated with COVID-19 mortality
Vitamin D Deficiency in Non-scarring and Scarring Alopecias: A Systematic Review and Meta-Analysis	Yongpisarn T, Tejapira K, Kunlawat Thadanipon K, Suchonwanit P.	Alopecias	Adults	Patients with non-scarring alopecia have insufficient serum VD level and increased incidence of vitamin D deficiency
Cardiometabolic factors and vitamin D deficiency in pediatric patients with chronic kidney disease	Fayoumi T, Gari A, Alarawi M, Almutairi S, Shalabi BH, Safdar O, Al Kadi H.	Chronic kidney disease (CKD)	Children	Children with CKD and low VD ( ≤ 41.9 nmol/L) presented a more adverse cardiometabolic risk profile
Relationship between Serum Vitamin D Levels and the Atherogenic Index of Plasma (AIP): A Study Based on NHANES Database 2011–2018	Hu T, Zhang Y, Chen Z, Su J.	Risk of atherosclerosis	Adults	A significant negative correlation and saturation effect was found between serum vitamin D and the atherogenic index of plasma (AIP) in a large general adult population (*n* = 9637) from the NHANES
Association between 25(OH) vitamin D and schizophrenia: shared genetic correlation, pleiotropy and causality	Rong G, G Li X, Lu H, Su M, Jin Y	Schizophrenia	Adults	NEK4 (associated with neuropsychiatric and substance use disorders) was identified as a new potential target of therapeutical interventions, evidencing the role of hyaluronan metabolism in the genetic association between 25(OH)D and schizophrenia
The effect of vitamin D supplementation on antibiotic use: a meta-analysis based on randomized controlled trials	Wang M, Wu Y, Xiang Z, Zhang Y, Huang T, Chen B	Antibiotic use	Adults	VD supplementation does not affect antibiotic use in the general population, but it may be beneficial in reducing antibiotic use among individuals < 70 years, with 25(OH)D < 75 nmol/L, or those suffering from respiratory tract infections.
A narrative review focusing on randomized clinical trials (RCTs) of vitamin D supplementation for COVID-19 disease	Huang L, Song Z	COVID-19	Adults	Available results on the effect on clinical benefit from VD supplementation in COVID-19 setting are still heterogeneous and sometimes conflicting
Vitamin D supplementation may be beneficial in improving the prognosis of patients with sepsis-associated acute kidney injury in the intensive care unit: a retrospective study	Sun J, Wang Y, Wang J, Wu H, Xu Z, Niu D	Sepsis-associated acute kidney injury	Adults	VD supplementation may be associated with a reduced mortality rate (in-hospital and at 28 and 90 days)
The effects of vitamin D levels on physical, mental health, and sleep quality in adults: a comprehensive investigation	Singh AK, Kumar S, Mishra S, Rajotiya S, Debnath S, Raj P, Bareth H, Singh M, Nathiya D, Tomar BS	Wellbeing (physical and mental health, and sleep quality)	Adults	A close direct association found a between higher VD levels and better physical and mental health

VD, vitamin D; VDR, vitamin D receptor; T2D, type 2 diabetes; BMI, body mass index; COPD, chronic obstructive pulmonary disease; KD, Kawasaki disease.

In any case, at present the high prevalence of vitamin D deficiency is a worldwide major public sanitary issue in every stage of life, including children and adolescents, where an inadequate status can have implications on future health and wellbeing (3, 4). In this regard, as many as five contributions focusing on different children issues are included in this Research Topic (see Table 1). Moreover, available dosing recommendations for vitamin D supplementation may considerably vary in the literature depending on the clinical setting and specific cohort evaluated. Importantly, there is still no international general consensus on how to define an optimal vitamin D status, mainly defined based on the inverse relationship of parathyroid hormone and 25(OH)D. For it concerns bone health, the Endocrine Society defines as adequate serum 25(OH)D levels higher than 75 nmol/L (30 ng/mL) and values higher than 50 nmol/L (20 ng/mL) as deficient (5); instead, the Institute of Medicine (IOM, now National Academy of Medicine) definition suggests vitamin D sufficiency for values higher than 50 nmol/L (20 ng/mL), insufficiency between 30 and 50 nmol/L (12–20 ng/mL); deficiency for levels lower than 30 nmol/L (12 ng/mL) (6). In addition, differences may also be due to methodological issues (e.g., seasonal sampling, variability in the vitamin D assays), characteristics of the studied populations, (e.g., age, sex, and calcium intake), and life-style habits (physical activity, outdoor activities, and sun exposure) (1). Moreover, the majority of evidence derived from studies conducted in adults, and even the threshold used to define vitamin D deficiency or insufficiency may not be appropriate when applied to children and/or adolescents. Indeed, currently, there are no specific guidelines and no clear consensus on targets for optimal vitamin D status and supplementation in most extra-skeletal conditions (1). Thus, for non-classical actions, more research is needed concerning the optimal 25(OH)D levels to maintain and clarify the suitability of 25(OH)D reference levels (in turn useful to decide the recommended vitamin D intake to maintain most people above this threshold, providing adequacy) and the role of vitamin D as key indicator of health in different clinical settings. We hope that manuscripts included in this Research Topic, which highlight the role and levels of vitamin D in different pathophysiological conditions, may contribute to the actual discussion in this field and may be appreciated by readers of Frontiers in Nutrition, facilitating further studies stimulated by the data reported and by the many challenges still to be solved in this important research area.

## References

[1] Della Nera G Sabatino L Gaggini M Gorini F Vassalle C. Vitamin D determinants, status, and antioxidant/anti-inflammatory-related effects in cardiovascular risk and disease: not the last word in the controversy. Antioxidants (Basel). (2023) 12:948. 10.3390/antiox12040948 37107323 PMC10135791

[2] Slominski AT Tuckey RC Jetten AM Holick MF. Recent advances in vitamin D biology: something new under the sun. J Investig Dermatol. (2023) 143:2340–2. 10.1016/j.jid.2023.07.003 37791933 PMC10841303

[3] Cui A Zhang T Xiao P Fan Z Wang H Zhuang Y. Global and regional prevalence of vitamin D deficiency in population-based studies from 2000 to 2022: a pooled analysis of 7.9 million participants. Front Nutr. (2023) 10:1070808. 10.3389/fnut.2023.1070808 37006940 PMC10064807

[4] Huh SY Gordon CM. Vitamin D deficiency in children and adolescents: epidemiology, impact and treatment. Rev Endocr Metab Disord. (2008) 9:161–70. 10.1007/s11154-007-9072-y 18175220

[5] Holick MF Binkley NC Bischoff-Ferrari HA Gordon CM Hanley DA Heaney RP. Evaluation, treatment, and prevention of vitamin D deficiency: an Endocrine Society clinical practice guideline. J Clin Endocrinol Metab. (2011) 96:1911–30. 10.1210/jc.2011-0385 21646368

[6] Ross AC Manson JE Abrams SA Aloia JF Brannon PM Clinton SK. The 2011 report on dietary reference intakes for calcium and vitamin D from the Institute of Medicine: what clinicians need to know. J Clin Endocrinol Metab. (2011) 96:53–8. 10.1016/j.jada.2011.01.004 21118827 PMC3046611

