# Corrigendum: Editorial: Vitamin D: from pathophysiology to clinical impact, volume II

**DOI:** 10.3389/fnut.2025.1574400

**Published:** 2025-03-13

**Authors:** Cristina Vassalle

**Affiliations:** Fondazione G Monasterio, Fondazione CNR-Regione Toscana G Monasterio, Pisa, Italy

**Keywords:** 25(OH)D, extraskeletal districts, reference levels, threshold, vitamin D

In the published article, an error was made. The incorrect editorial had been submitted to Volume I of this Research Topic. This incorrect editorial has been transferred to the correct volume and further contributions have been added to editorial titled “Vitamin D: From Pathophysiology to Clinical Impact Volume II”. These contributions appear below.

**Table:** 

Association between 25(OH) vitamin D and schizophrenia: shared genetic correlation, pleiotropy and causality	Rong G, G Li X, Lu H, Su M, Jin Y	Schizophrenia	Adults	NEK4 (associated with neuropsychiatric and substance use disorders) was identified as a new potential target of therapeutical interventions, evidencing the role of hyaluronan metabolism in the genetic association between 25(OH)D and schizophrenia
The effect of vitamin D supplementation on antibiotic use: a meta-analysis based on randomized controlled trials	Wang M, Wu Y, Xiang Z, Zhang Y, Huang T, Chen B	Antibiotic use	Adults	VD supplementation does not affect antibiotic use in the general population, but it may be beneficial in reducing antibiotic use among individuals < 70 years, with 25(OH)D < 75 nmol/L, or those suffering from respiratory tract infections.
A narrative review focusing on randomized clinical trials (RCTs) of vitamin D supplementation for COVID-19 disease	Huang L, Song Z	COVID-19	Adults	Available results on the effect on clinical benefit from VD supplementation in COVID-19 setting are still heterogeneous and sometimes conflicting
Vitamin D supplementation may be beneficial in improving the prognosis of patients with sepsis-associated acute kidney injury in the intensive care unit: a retrospective study	Sun J, Wang Y, Wang J, Wu H, Xu Z, Niu D	Sepsis-associated acute kidney injury	Adults	VD supplementation may be associated with a reduced mortality rate (in-hospital and at 28 and 90 days)
The effects of vitamin D levels on physical, mental health, and sleep quality in adults: a comprehensive investigation	Singh AK, Kumar S, Mishra S, Rajotiya S, Debnath S, Raj P, Bareth H, Singh M, Nathiya D, Tomar BS	Wellbeing (physical and mental health, and sleep quality)	Adults	A close direct association found a between higher VD levels and better physical and mental health

The author apologizes for this error and states that this does not change the scientific conclusions of the article in any way. The original article has been updated.

